# Evaluation of SBDP145, melatonin, sLOX-1, HMGB1 and HIF-1α in preterm infants with brain injury

**DOI:** 10.1186/s13052-024-01744-4

**Published:** 2024-09-11

**Authors:** Sisi Cheng, Xiao Sun, Yanyan Li, Yan Dong

**Affiliations:** Department of Pediatrics, The First Hospital of Hebei Medical University, No. 89 Donggang Road, Yuhua District, Shijiazhuang, Hebei 050000 China

**Keywords:** SBDP145, Melatonin, sLOX-1, Brain injury, Premature infants, Prognosis

## Abstract

**Background:**

Prematurity-related brain injury is a common and serious complication that has long-term effects on the survival and development of affected infants. Currently, the roles of certain biomarkers such as the protein hydrolysis product SBDP145, melatonin, soluble lectin-like oxidized low-density lipoprotein receptor-1 (sLOX-1), high mobility group box 1 protein (HMGB1), and hypoxia-inducible factor 1-alpha (HIF-1α) in prematurity-related brain injury remain not fully elucidated. Our study aims to assess the significance of SBDP145, melatonin, sLOX-1, HMGB1 and HIF-1α in preterm infants with brain injury.

**Methods:**

135 preterm infants admitted to our hospital from January 2020 to February 2022 were selected and divided into 78 cases in a prematurity-associated brain injury group, and 57 cases in another group of preterm infants without brain injury or other diseases according to the magnetic resonance imaging results. The levels of SBDP145, melatonin, sLOX-1, HMGB1 and HIF-1α in the two groups were analyzed. The serum concentrations of SBDP145, melatonin, sLOX-1, HMGB1 and HIF-1α in newborns with different severity of ventricular hemorrhage were observed, and the levels of SBDP145, melatonin, sLOX-1, HMGB1 and HIF-1α in those with different severity of white matter brain injury were compared.

**Results:**

The levels of SBDP145, sLOX-1, HMGB1 and HIF-1α were significantly higher in the preterm combined brain injury group than in the preterm group, and melatonin levels were significantly lower than in the preterm group(*P* < 0.05). The levels of SBDP145, sLOX-1, HMGB1 and HIF-1α were higher in the moderate to severe group and melatonin levels were lower in the mild group of newborns with ventricular hemorrhage (*P* < 0.05). The levels of SBDP145, sLOX-1, HMGB1 and HIF-1α were higher in the moderate-severe group and melatonin levels were lower in the mild group in newborns with cerebral white matter injury (*P* < 0.05). The independent variables were SBDP145, melatonin, sLOX-1, HMGB1, HIF-1α, and the dependent variable was the prognosis of neonates with brain injury. Univariate logistic regression analysis and multivariate logistic regression analysis were performed. The results showed that the influencing factors of newborns with brain injury were SBDP145, melatonin, sLOX-1, HMGB1, HIF-1α.

**Conclusion:**

The levels of SBDP145, melatonin, sLOX-1, HMGB1 and HIF-1α were highly expressed in preterm newborns with brain injury, and the levels were higher when the condition of the newborns was more severe. These findings suggest the potential clinical utility of these biomarkers in predicting and monitoring brain injury in preterm infants, which could aid in early intervention and improve long-term outcomes.

## Introduction

The incidence of premature infants in China is about 8 -10%. About 1.2–1.5 million premature infants are born every year, including about 300,000 premature ones with a gestational age of less than 32 weeks [1]. The survival rate of those born < 32 weeks of gestation increased by 22.7% from 2012 to 2017, reaching 63.6%, and that of those weighting < 1000 g was about 42.3% [2]. Despite the high survival rate of preterm infants, the incidence of brain injury in this population has not decreased [3]. Brain injury in premature infants refers to cerebral ischemia or hemorrhagic damage caused by various pathological factors before, during, and after birth. These factors can result in different degrees of damage to the neurological function of children in the later period, and even endanger their lives [4]. Premature-related cerebral damage according to pathology can be divided into white matter injury, and non-parenchymal, brain parenchyma and cerebellum brainstem hemorrhage [5]. The causes of brain injury in premature infants mainly include immune inflammatory response caused by ischemia and hypoxia, infection or tissue damage, which affect the precursor cells of oligodendrocytes and the maturation and myelination of neurons [5]. In addition, fragility of the vascular germinal matrix has also been proposed as an important cause of brain injury in preterm infants [6]. However, the pathophysiology of cerebral damage in these neonates is complex and still not fully elucidated. Early prediction and diagnosis of brain injury and active intervention can greatly improve the quality of life and reduce the disability and mortality of patients.

Spectrin breakdown product 145(SBDP145) is an important component of α II spectrin [7]. When axonal damage occurs, αII spectrin in the damaged brain cells decomposes into SBDP145, which can enter the peripheral blood through the blood-brain barrier [8, 9]. SBDP145 levels are highly expressed in the serum of children with traumatic brain injury and hypoxic-ischaemic encephalopathy [10]. Melatonin is a metabolite of L-tryptophan, mainly synthesized by pineal cells, and is associated with neurological disease [11, 12]. sLOX-1 is a major molecular fragment of soluble lectin-like oxidized LDL receptor-1 (LOX-1) and plays a role in the vascular inflammatory response and atherosclerosis [13, 14]. HMGB1 is a highly conserved DNA-binding protein present in brain cells, and involved in the development of atherosclerosis [15, 16]. HMGB1 plays an important role in the pathogenesis of acute lung injury, idiopathic pulmonary fibrosis, acute pancreatitis, sepsis, and inflammatory bowel disease [17–19]. Its levels are elevated in hypoxic-ischemic encephalopathy, and may be an indicator of early inflammation [20, 21]. However, a few studies on HMGB1 and preterm infants with brain injury are available. HIF-1α is a hypoxic transcription factor that participates in the regulatory process of hypoxic injury in the body and promotes the reestablishment of microcirculation and anaerobic metabolism in order to protect brain tissue [22]. These biomarkers may play a crucial role in brain injury among premature infants because they represent distinct pathophysiological processes or inflammatory responses. Investigating their correlation with brain injury in preterm infants can provide deeper insights into the pathogenesis of the disease, thereby offering novel biomarker indicators for early prediction, diagnosis, and intervention. Hence, exploring the relationships between SBDP145, melatonin, sLOX-1, HMGB1, and HIF-1α and brain injury in premature infants holds significant clinical and scientific relevance. On this basis, this study will investigate the correlation of SBDP145, melatonin, sLOX-1, HMGB1 and HIF-1α in preterm infants suffering from brain injury.

## Methods

The 135 preterm infants (defined as those who are up to 28 weeks of gestational age but less than 37 weeks) admitted to our hospital from January 2020 to February 2022 were selected. According to the magnetic resonance imaging findings, 78 cases were included into a preterm with brain injury group and 57 subjects into a preterm without brain involvement group. Brain injury included white matter brain injury and ventricular hemorrhage. All protocols were approved by the local ethical committee of the First Hospital of Hebei Medical University. Informed consent was obtained from all the patients’ legal guardian(s). The study protocol was in accordance with the requirements of the Declaration of Helsinki [23].

### Inclusion and exclusion criteria

Inclusion criteria:

(i) singleton pregnancy;
(ii) undergoing cranial MRI (Magnetic Resonance Imaging);
(iii) admitted within 6 h of birth;
(iv) discharged for outpatient follow-up;
(v) presence of high risk factors for neonatal brain injury: including hypothermia, intrauterine growth restriction, haemodynamic disturbances, hypercapnia or hypocapnia, respiratory failure, presence of infection and inflammatory response, moderate to severe anaemia, coagulation abnormalities.
(vi) all parents gave informed consent.

### Exclusion criteria

(i) serious complications during pregnancy affecting gestation outcome;
(ii) neurological infections, hypoglycaemic encephalopathy or bilirubin encephalopathy during hospitalisation;
(iii) congenital neurological malformations, comorbidities including genetic metabolic disorders;
(iv) unwillingness to participate in this study;
(v) incomplete information such as transfer to other hospital during treatment, or its abandonment.

### Diagnostic and grading criteria for brain injury in preterm infants

Premature infants received an initial cranial ultrasound examination 24 h after birth, followed by weekly follow-up until discharge, and a cranial MRI examination before discharge or at 40 weeks of corrected gestational age; in newborns with suspected intracranial pathology but normal ultrasound, a cranial MRI examination was performed to further determine the presence of brain injury. Types of brain injury included ventricular haemorrhage and white matter damage. Ventricular haemorrhage was graded according to the Papile classification [24]. Grade I-II for mild injury; Grade III-IV for moderate to severe injury. Grading criteria for cerebral white matter injury [25]: mild injury: Grade 0–1; moderate to severe injury: Grade 2–3.

### General information

The gender, gestational age, delivery mode, birth weight, 1 min Apgar score and 5 min Apgar score of all subjects were collected.

### Indicator tests

1 ml of peripheral venous blood was collected from all study subjects within 3 days of birth, centrifuged at 3500r/min (10 cm radius) for 15 min, and the supernatant was aspirated for testing. Serum SBDP145, melatonin, sLOX-1, HMGB1 and HIF-1α levels were measured by ELISA.

### Prognostic follow-up

All subjects were followed up once a month for 6 months and every 3 months after 6 months. At 9 months of age, infants were assessed using the Bayley Scales of Infant and Toddler Development (BSID-II) for motor development index (PDI) and mental development index (MDI), with normal development being defined as a score of ≥ 70 on both the PDI and MDI, and abnormal development being defined as a score of < 70 on either scale.

### Statistics

SPSS 21.0 software was used to analyze the data. The measurement data conforming to the normal distribution were expressed as mean ± SD. The overall comparison of each group of data was analyzed by one-way ANOVA, and the pairwise comparison of data between groups and within groups was analyzed by LSD method. Count data were expressed as rate (%) and compared by chi-square test; Pearson was used to analyze correlations; univariate / multivariate logistic regression was used to analyze the influencing factors of premature infants with brain injury. *P* < 0.05 was considered significant.

## Results

### Comparison of general information between the two groups

There were 78 newborns in the preterm with brain injury group, of whom 47 were male and 31 were female. In the group of preterm infants, there were 57 subjects, including 31 males and 26 females. The differences in gender, mode of delivery, birth weight, 1 min Apgar score and 5 min Apgar score between the two groups were not significant (*p* > 0.05) as shown in Table 1.

**Table 1:** Comparison of general information between the two groups

Item	Premature infants with brain injury (*n* = 78)	Premature infants without brain injury (*n* = 57)	χ^2^/t	*P*
Gender (male/female,%) (n)	47/31 (60.26%/39.74%)	31/26 (54.39%/45.61%)	0.465	0.495
Gestational age (weeks)	35.12 ± 1.22	35.01 ± 2.15	0.377	0.707
Mode of delivery (n)			1.071	0.301
Caesarean section,%	48(61.54%)	30(52.63%)		
Normal delivery,%	30(38.46%)	27(47.37%)		
Birth weight (kg)	2.034 ± 0.341	2.054 ± 0.425	-0.004	0.997
Apgar score at 1 min	8.71 ± 0.83	8.78 ± 0.89	-0.621	0.576
Apgar score at 5 min	9.03 ± 0.65	9.12 ± 0.69	-0.774	0.445
Ventricular haemorrhage (n)				
Mild injury,%	27(34.46%)	-		
Moderate to severe injury,%	19(24.36%)	-		
Brain white matter injury (n)				
Mild injury,%	20(25.64%)	-		
Moderate to severe injury,%	12(15.38%)	-		
PDI score	70.23 ± 4.12	69.38 ± 4.19	1.178	0.241
MDI score	70.34 ± 3.09	69.47 ± 3.07	1.619	0.108

### Comparison of SBDP145, melatonin, sLOX-1, HMGB1 and HIF-1α levels between the two groups

The levels of SBDP145, sLOX-1, HMGB1 and HIF-1α in the group of preterm infants with brain injury were higher than those in the preterm infant group, and melatonin levels were lower than those in the preterm infant group, with significant differences (*P* < 0.05), as shown in Table 2.

**Table 2:** Comparison of SBDP145, melatonin, sLOX-1, HMGB1 and HIF-1α levels between the two groups

Item	Premature infants with brain injury (*n* = 78)	Premature infants without brain injury (*n* = 57)	t	*P*
SBDP145(ng/ml)	2.67 ± 0.37	1.29 ± 0.32	22.369	<0.001
Melatonin(pg/ml)	871.91 ± 10.23	3468.29 ± 13.29	-1282.594	<0.001
sLOX-1(pg/ml)	413.98 ± 23.29	376.91 ± 25.12	8.835	<0.001
HMGB1(ng/ml)	79.21 ± 4.91	58.91 ± 4.78	419.644	<0.001
HIF-1α(ng/ml)	1.38 ± 0.29	0.39 ± 0.11	24.498	<0.001

### Comparison of SBDP145, melatonin, sLOX-1, HMGB1 and HIF-1α levels in newborns with different severity of ventricular haemorrhage

Newborns with ventricular haemorrhage were divided into a moderate to severe group (as shown in Table 1, *n* = 19) and a mild group (*n* = 27). The levels of SBDP145, sLOX-1, HMGB1 and HIF-1α were found to be higher in the moderate-severe group than in the mild group, and melatonin levels were lower among the former than in the mild group, with significant differences (*P* < 0.05), as shown in Table 3.

**Table 3:** Comparison of SBDP145, melatonin, sLOX-1, HMGB1 and HIF-1α levels in infants with different severity of ventricular hemorrhage

Item	Mild (*n* = 27)	Moderate to severe (*n* = 19)	t	*P*
SBDP145(ng/ml)	2.32 ± 0.41	2.98 ± 0.45	-8.864	<0.001
Melatonin(Pg/ml)	1087.65 ± 12.87	632.32 ± 13.92	196.141	<0.001
sLOX-1(pg/ml)	401.03 ± 23.77	421.87 ± 25.21	-4.904	<0.001
HMGB1(ng/ml)	71.29 ± 5.09	87.93 ± 5.03	-18.854	<0.001
HIF-1α(ng/ml)	1.12 ± 0.21	1.83 ± 0.22	-19.016	<0.001

Note: Grade I-II for mild injury; Grade III-IV for moderate to severe injury

### Comparison of the levels of SBDP145, melatonin, sLOX-1, HMGB1 and HIF-1α in newborns with different severity of brain white matter injury

Newborns with cerebral white matter injury were divided into a moderate to severe group (as shown in Table 1, *n* = 12) and a mild group (*n* = 20), and the levels of each index were compared between the two groups. The levels of SBDP145, sLOX-1, HMGB1 and HIF-1α were found to be higher in the moderate-severe group than in the mild group, and the levels of melatonin were lower in the first group than in the mild one, with significant differences (*P* < 0.05), as shown in Table 4.

**Table 4:** Comparison of SBDP145, melatonin, sLOX-1, HMGB1 and HIF-1α levels in infants with different severity of brain white matter injury

Item	Mild (*n* = 20)	Moderate to severe (*n* = 12)	t	*P*
SBDP145(ng/ml)	2.37 ± 0.39	2.91 ± 0.42	-7.691	<0.001
Melatonin(pg/ml)	1081.29 ± 14.01	645.39 ± 14.09	178.124	<0.001
sLOX-1(pg/ml)	403.12 ± 24.02	420.21 ± 25.09	-4.007	<0.001
HMGB1(ng/ml)	71.68 ± 6.08	86.83 ± 6.81	13.591	<0.001
HIF-1α(ng/ml)	1.16 ± 0.23	1.81 ± 0.25	-15.632	<0.001

Note: Mild injury: Grade 0–1; moderate to severe injury: Grade 2–3

### Univariate Logistic Regression Analysis of Risk factors for newborns with Brain Injury

The results showed that the factors influencing the prognosis of neonates with brain injury were SBDP145, melatonin, sLOX-1, HMGB1 and HIF-1α, see Table 5.

**Table 5:** Univariate logistic regression analysis of risk factors for prognosis of neonates with brain injury

Variables	β	SE	Wald	OR(95%CI)	*P*
SBDP145	2.156	0.965	4.871	3.291(1.376–6.981)	0.023
Melatonin	0.119	0.053	5.237	1.129(1.012–1.654)	0.019
sLOX-1	0.019	0.013	4.387	1.027(1.001–1.239)	0.039
HMGB1	1.115	0.329	11.092	3.001(1.673–5.876)	0.001
HIF-1α	0.583	0.182	10.482	1.746(1.276–2.602)	<0.001

### Multi-factor logistic regression analysis of risk factors for prognosis of neonates with brain injury

The results showed that the factors influencing the prognosis of neonates with brain injury were SBDP145, melatonin, sLOX-1, HMGB1 and HIF-1α, see Table 6.

**Table 6:** Multifactorial logistic regression analysis of risk factors for prognosis of neonates with brain injury

Variables	β	SE	Wald	OR(95%CI)	*P* value
SBDP145	1.037	0.362	8.198	2.781(1.281–4.391)	0.003
Melatonin	1.159	0.429	7.238	3.091(1.367–7.981)	0.007
sLOX-1	1.029	0.402	6.128	2.651(1.198–5.187)	0.013
HMGB1	1.019	0.653	2.651	2.771(1.428–4.871)	<0.001
HIF-1α	0.978	0.691	1.912	2.472(1.327–5.081)	<0.001

## Discussion

Biomarkers can raise early alarms for the body by changing at the molecular and cellular levels before serious damage occurs. The results of this study showed that the levels of SBDP145, sLOX-1, HMGB1 and HIF-1α in the group of preterm infants with brain injury were higher than that of premature newborns without brain injury, and the level of melatonin was lower than that in the premature infant group (*P* < 0.05).

Brain injury in preterm infants is predominantly associated with ventricular haemorrhage and periventricular white matter softening. In this study, approximately 58.97% (46/78) of ventricular haemorrhages and 41.03% (32/78) of periventricular white matter softening were observed. The levels of SBDP145, sLOX-1, HMGB1 and HIF-1α were higher in the moderate-severe group than in the mild group (*p* < 0.05). It is suggested that the more severe the degree of brain damage in preterm infants, the higher the levels of SBDP145, sLOX-1, HMGB1 and HIF-1α. Melatonin was found to have strong antioxidant capacity and could reduce the size of cerebral infarcts and brain edema, thus exerting a cerebral protective effect [26]. We found that melatonin levels were significantly lower in the group with brain injury compared to that without, and that its levels were lower in the group with ventricular hemorrhage of moderate to severe degree, compared to the mild degree group. These results suggest that the antioxidant capacity of melatonin may reduce the severity of cerebral infarction.

Mondello et al. [10] pointed out that serum SBDP145 levels could be used to assess the severity of brain injury and determine prognosis in patients with spontaneous cerebral haemorrhage. In a study, sLOX-1 levels were highly expressed in newborns with hypoxic-ischemic encephalopathy and could be used to assess the severity of the disease [27]. HMGB1 was shown to be a useful factor in the prognosis of newborns with hypoxic-ischaemic encephalopathy [28]. When a stroke injury occurs, HMGB1 enters the bloodstream to promote an inflammatory response and exacerbate brain injury. It has been suggested that serum HMGB1 levels are highly expressed after severe preterm brain injury [29]. HIF-1α levels are highly expressed in preterm infants with brain injury [22]. Li found that the higher the level of HIF-1α, the more severe the brain injury [30]. The results of this study showed that after single and multiple factor logistic regression analysis, SBDP145, melatonin, sLOX-1, HMGB1 and HIF-1α could be used as influential factors in assessing the prognosis of neonates with brain injury. These results lead us to consider whether these biomarkers could also serve as prognostic factors in other neonatal neuro-related diseases, such as neural tube defects or genetic diseases/malformation syndromes [31–33].

This study also has several limitations. The levels of SBDP145, melatonin, sLOX-1, HMGB1, and HIF-1α in newborns with preterm brain injury were not dynamically analyzed, and further investigation into the dynamic changes of these markers is warranted. Additionally, hypothermia, which may temporarily impair the neonatal immune system [34], could potentially affect the levels of these markers, thereby influencing the results. Lastly, all newborns in this study were from our hospital, and it remains uncertain whether geographical differences and dietary habits may have influenced the results. Further research will be conducted to address these aspects.

## Conclusion

SBDP145, sLOX-1, HMGB1, and HIF-1α levels were found to be significantly elevated in preterm newborns with brain injury. Moreover, the severity of the child’s condition was found to be positively correlated with increased levels of these markers. SBDP145, melatonin, sLOX-1, HMGB1, and HIF-1α can be regarded as indicative markers for diagnosing brain injury in preterm infants. These markers serve as valuable adjuncts to neonatologists, assisting them in providing more comprehensive prognostic evaluations of brain injury by complementing clinical features, laboratory results, and instrumental findings [35, 36].

## Data Availability

The datasets used and analysed during the current study can be available from the corresponding author on reasonable request.

## Abbreviations

HIF-1α: Hypoxia inducible factor-1α
HMGB1: High mobility group box 1
SBDP145: Spectrin breakdown product 145
sLOX-1: Soluble lectin-like oxidized LDL receptor-1
